# Measuring Plant Cell Wall Extension (Creep) Induced by Acidic pH and by Alpha-Expansin

**DOI:** 10.3791/1263

**Published:** 2009-03-11

**Authors:** Daniel M. Durachko, Daniel J. Cosgrove

**Affiliations:** Department of Biology, University of Pennsylvania

## Abstract

Growing plant cell walls characteristically exhibit a property known as 'acid growth', by which we mean they are more extensible at low pH (< 5) ^1^. The plant hormone auxin rapidly stimulates cell elongation in young stems and similar tissues at least in part by an acid-growth mechanism ^2, 3^. Auxin activates a H^+^ pump in the plasma membrane, causing acidification of the cell wall solution. Wall acidification activates expansins, which are endogenous cell wall-loosening proteins ^4^, causing the cell wall to yield to the wall tensions created by cell turgor pressure. As a result, the cell begins to enlarge rapidly. This 'acid growth' phenomenon is readily measured in isolated (nonliving) cell wall specimens. The ability of cell walls to undergo acid-induced extension is not simply the result of the structural arrangement of the cell wall polysaccharides (e.g. pectins), but depends on the activity of expansins ^5^. Expansins do not have any known enzymatic  activity and the only way to assay for expansin activity is to measure their induction of cell wall extension. This video report details the sources and preparation techniques for obtaining suitable wall materials for expansin assays and goes on to show acid-induced extension and expansin-induced extension of wall samples prepared from growing cucumber hypocotyls.

To obtain suitable cell wall samples, cucumber seedlings are grown in the dark, the hypocotyls are cut and frozen at -80 °C.  Frozen hypocotyls are abraded, flattened, and then clamped at constant tension in a special cuvette for extensometer measurements.  To measure acid-induced extension, the walls are initially buffered at neutral pH, resulting in low activity of expansins that are components of the native cell walls. Upon buffer exchange to acidic pH, expansins are activated and the cell walls extend rapidly. We also demonstrate expansin activity in a reconstitution assay. For this part, we use a brief heat treatment to denature the native expansins in the cell wall samples. These inactivated cell walls do not extend even in acidic buffer, but addition of expansins to the cell walls rapidly restores their ability to extend.

**Figure:**
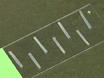


## Protocol

### Part 1: Growing and storing suitable plant material

In our experience, young hypocotyls from etiolated cucumber seedlings serve as a convenient source of cell wall material for these experiments. Cucumber seeds are sown on wet paper in a light-proof box, which is keep in a darkened cabinet in a constant temperature room set at 26 °C.  The exact temperature is not critical, as anything between 22° and 30 °C  should be fine, but the temperature will determine how fast the seedlings reach an appropriate stage of development. The warmer the temperature, the faster the seedlings will develop. We typically use seedlings when they have grown to about 5 cm in length, which is reached 3-4 days after sowing. It is important that the seedling be grown in the dark, as even small amounts of  light affect both the rate of seedling development and the cell wall properties that we measure with this technique.  On day 3 you can peek in the box, using a dim green filtered light, to check on seedling development.
Seedlings are rapidly cut and packed in small plastic boxes, 100-150 seedlings per box, and stored at -80 °C. At this temperature they remain useful for weeks.

### Part 2: Preparing cell wall samples

Small groups (8-10) of frozen cut seedlings are transferred from the freezer to an insulated container containing a -80 freezer block.
The cuticle covering the hypocotyl is abraded with carborundum. This is done by repeatedly drawing the hypocotyl between the thumb and forefinger, which are coated with a thick slurry of wet carborundum powder. It takes a little experience to know the correct amount of pressure to use: too much pressure and the epidermal layer begins to be shredded and torn; too little pressure and the cuticle will not be permeabilized.  One also has to work quickly because as the frozen hypocotyl thaws, it becomes flaccid and hard to manage.
The abraded hypocotyl is dipped in ice water to remove most of the adhering carborundum and then stored on ice water while the remaining hypocotyls are prepared in a similar manner.
The hypocotyls are cut to the desired length, usually 1.2 cm, with a new single edged razor blade and then aligned on a glass slide.
Now we need to flatten the walls to remove cell sap and to facilitate clamping. A second glass slide is placed on top of the group of 8-10 samples, forming a sandwich. A weight (400-500 g) is placed on top of the glass slide for 5 min.  For the weight we routinely use a beaker containing a suitable amount of water.
Optional step:  Depending on the experiment, the hypocotyls may be inactivated with a brief heat treatment at this point. To do this we bind the glass slides together with a pair of rubber bands, put the assembly in a container with 100 mL of de-ionized water at room temperature and place it in a microwave oven at full power.  With our microwave oven the water starts to boil at about 50 s and we stop the microwave  15 s after boiling begins.  The hot water is quickly poured off and replaced with cold water to stop denaturation.  You may have to vary these timings, as your microwave may be different than ours. With excessive heating the samples become weak and break easily. With insufficient heating the endogenous expansin is not inactivated and the wall samples retain some responsiveness to acidic pH.

### Part 3: Extensometer setup

The wall samples are now clamped in a constant force extensometer. This is a custom-built device that consists of a Plexiglas cuvette for holding the basal end of the wall sample and a moveable clamp attached to the apical end of the sample. The moveable clamp is mounted on the end of a rod that passes through the open coils of a position sensor, an LVDT or 'Linear Variable Differential Transformer', that electronically detects the position of a small metal cylinder, or 'core', that is attached to the rod.  The upper end of the rod is linked to a lever with an adjustable counterweight. This lever exerts an adjustable amount of upward force on the wall sample.  The force is adjusted by adding or removing calibrated metal weights to the far end of the lever.
Back to the wall sample - the basal end of the hypocotyl sample is picked up with fine forceps and ~2-3 mm of the apical end is placed in between the open jaws of the moveable clamp. This clamp is a spring-loaded alligator clamp whose metal  jaws are coated with plastic to prevent direct contact between the metal surface and the buffer solution or the wall sample. In our experience metal ions can leach from the clamp and inhibit the wall's ability to extend, and so we keep the metal covered.
Holding the moveable clamp assembly in one hand, the basal end of the wall specimen is now maneuvered in between the two hinged pieces of the Plexiglas cuvette and the cuvette pieces are brought together and screwed tight, thereby locking the bottom end of the wall sample in the cuvette.
The moveable clamp assembly is now gently  released, allowing the full force of the counterweights to be transferred to the wall specimen.  We routinely use a total counterweight of 20 g for wall samples prepared from cucumber hypocotyls. Other wall materials or experiments might require different weights.
The cuvette is filled with buffer (200 uL) and the position of the cuvette moved up or down with an adjustment screw, so that the moveable clamp is brought to the lower end of the LVDT measuring range. Our LVDT is connected to a data acquisition unit of a microcomputer, and so we monitor LVDT position through the computer.  We have eight LVDT assemblies connected in parallel with one computer, which allows us to run 8 wall samples simultaneously.  The computer records the position of each of the LVDT assemblies once every 30 s.

### Part 4: Measuring extension response to acid pH or expansin - Representative Results

**[First Experiment]** For measurement of acid-induced extension, we start with native wall samples (that is, not inactivated with heat) and neutral buffer is added to the cuvette.  The wall samples extend for a few minutes, in response to the added tension, but the extension decays to a low rate after a few minutes. Our computer lets us monitor either the change in length of the sample (that is, the change in position of the moveable clamp, after the start of the experiment) or we can monitor the time derivative of position, in other words the rate of extension. The extension rate stabilizes to a low value with time.
After ~20 min, the neutral buffer is removed. We use a thin metal pipe, made from a large gauge hypodermic needle,  connected to a vacuum pump to remove the buffer quickly, with minimal disturbance of the wall or the mechanical assembly. Acidic buffer is then added, sometimes with 1-2 quick exchanges to insure the complete exchange to acidic buffer. Then we sit back and watch the response of the wall. Typically we can detect the faster rate of extension in a few minutes.  After 60 min we usually have enough information to assess the extension response, although in some cases the measurements may extend to longer periods.
**[Second Experiment]** For measurement of expansin-induced cell wall extension, we start with heat-inactivated wall samples and acidic buffer is added to the cuvette. As in the First Experiment, the rate of cell wall extension gradually stabilizes to a low value because of the lack of functional expansin.
After ~20 min, expansin protein is added to the cuvette by 'spiking' with the buffer in the cuvette with 10-20 uL of an expansin solution. The expansin rapidly penetrates the cell wall sample and within a few minutes we see that the extension rate has increased.  This extension response can be followed for an hour or more.

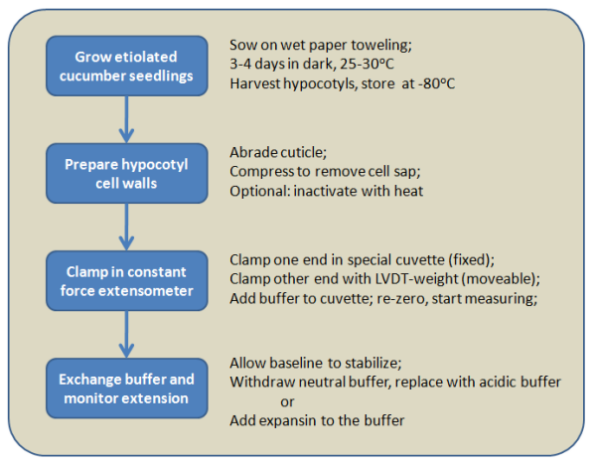
 **Figure 1:** Outline of procedures for preparing  cell walls to assess acid-induced or expansin-induced wall extension.

## Discussion

For this demonstration we used cell walls from cucumber hypocotyls because they have proved to be a reliable source of wall samples that are easy to handle and that respond with good sensitivity.  We have also had good success with walls from other seedlings as well as some materials from the supermarket, such as young spinach leaves and celery stalks. Basically, young, soft, rapidly growing plant tissues are likely to be easily measured with this technique, but tough, old, oxidized or nongrowing plant tissues are unlikely to be responsive because of cell wall cross linking. There are a few other things to beware of:

Biological variability - even uniform seedlings give variable responses, so a minimum of 5-8 replicates is necessary to yield statistically meaningful results.
Abrasion of the cuticle is important to permit rapid penetration of buffers and proteins into the cell wall specimen. If the cuticle is not sufficiently abraded, responses to acidic buffers will be slow and muted and proteins may not even penetrate the cuticle to elicit a response. Some samples might not need abrasion, i.e. if you use epidermal peels or other dissected tissues. Nonuniform abrasion adds significant variability for many novices.
Heat inactivation, which is used  to remove or denature endogenous expansins, can be carried out by other methods, but one needs to find the minimal amount of heating to inactivate endogenous expansin to avoid excessive weakening and breakage of the wall samples.
Expansins are prone to inactivation by oxidation, so 1-5 mM dithiothreitol in the buffer usually helps to stabilize activity.
The extensometer is not an off-the-shelf piece of equipment, but requires custom construction of (a) the cuvette that holds the wall specimen and (b) the LVDT-clamp-counterweight assembly. The computer interface and data acquisition unit are not essential, but are particularly valuable for running replicate samples simultaneously and for analyzing the extension curves quantitatively.
